# Nodules de Lisch: marqueur ophtalmologique de la neurofibromatose de type 1

**DOI:** 10.11604/pamj.2022.42.108.35079

**Published:** 2022-06-09

**Authors:** Kawtar Bouirig, Lalla ouafae Cherkaoui

**Affiliations:** 1 Ophthalmology Department “A”, Ibn Sina University Hospital (*Hôpital des Spécialités*), Mohammed V University, Rabat, Morocco

**Keywords:** Dermatologie, nodules de Lisch, maladie de Von Recklinghausen, neurofibromatose de type I, dermatology, Lisch nodules, Von Recklinghausen disease, neurofibromatosis type I

## Abstract

Von Recklinghausen disease or neurofibromatosis type I (NF1) is the most common phacomatosis. It is inherited in an autosomal dominant manner. Lisch nodules are the most common ophthalmic manifestation of NF1. They are reported in 73-95% of cases. We here report the case of a 49-year-old female patient referred for a consultation with an ophthalmologist for routine eye check-up after the onset of the following symptoms: coffee-with-milk colored spots and multiple neurofibromas (A). She reported similar family history. Visual acuity was 10/10 P2 in both eyes. Examination of the anterior segment showed several Lisch nodules in both irises. They were scattered over the entire iris surface and varied in size (B). Fundus examination was unremarkable. CT scan of the brain and the orbit as well as thoracoabdominal CT scan showed no associated lesion. The patient met NIH diagnostic criteria for NF1. Lisch nodules are small brown dome-shaped lesions developing on the surface of the iris, with well-defined edges, embedded in the stroma and lighter than iris pigmentation. Differential diagnosis includes iris mammillations, iris naevi, iris melanoma, iris granulomatous nodules. Unlike skin signs, the presence of multiple Lisch nodules are considered a specific indication of NF1. These nodules may occur in childhood and their prevalence and number increase with age. Thus, periodic eye examinations in subjects with suspected neurofibromatosis type I may help to improve early diagnosis.

## Image en médecine

La maladie de Von Recklinghausen ou neurofibromatose de type I (NF1) est la plus fréquente des phacomatoses. Les formes héréditaires sont transmises sur un mode autosomique dominant. Les nodules de Lisch sont la manifestation ophtalmologique la plus courante de la NF1, rapportée dans 73 à 95% des cas. Nous rapportons le cas d´une patiente de 49 ans, adressée en consultation d´ophtalmologie pour bilan oculaire systématique devant les signes cutanés suivants: taches café au lait et neurofibromes multiples (A). L'interrogatoire retrouve la notion d'antécédents familiaux similaires. L´acuité visuelle était de 10/10 P2 aux deux yeux. L´examen du segment antérieur montre de nombreux nodules de Lisch sur les deux iris. Ils sont disséminés sur l'ensemble de la surface irienne, de tailles variables (B). Le fond d'œil est sans particularité. Le scanner orbito-cérébral et thoraco-abdominal n´ont révélé aucune lésion associée. La patiente répond aux critères diagnostiques NIH de la NF1. Les nodules de Lisch sont de petites lésions surélevées par rapport à la surface de l'iris, à bord très net, comme enchâssées dans le stroma, plus claires que la pigmentation de l'iris, brunes. Le diagnostic différentiel inclue les mamelonnements de l´iris, naevi de l´iris, mélanome de l´iris,nodules granulomateux iriens . Contrairement aux signes cutanés, plusieurs nodules de Lisch sont considérés comme spécifiques de la NF1. Ces nodules peuvent apparaître tôt dans l´enfance et leur prévalence et leur nombre augmentent avec l´âge. Ainsi, des examens ophtalmologiques périodiques chez des individus suspects peut aider au diagnostic clinique précoce de la NF1.

**Figure 1:**
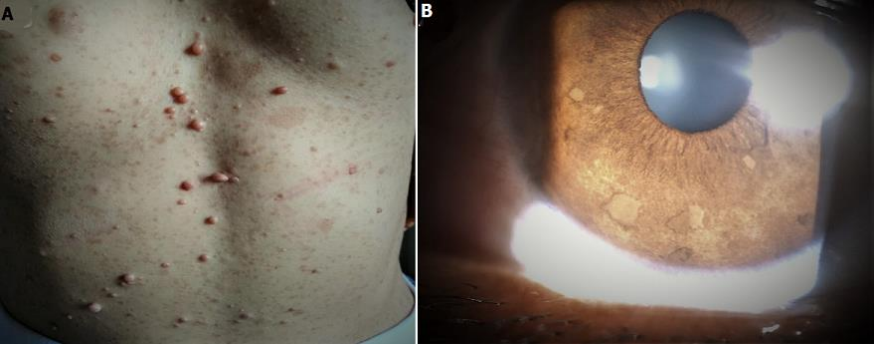
A) photographie du tronc montrant de multiples taches café au lait et neurofibromes; B) nodules multiples hyperpigmentés de taille variable sur la face antérieure de l´iris évoquant des nodules de Lisch notés à l´examen à la lampe à fente

